# From Surviving to Excelling: Adaptation of Gerontology Service Learning During the COVID-19 Pandemic

**DOI:** 10.1093/geroni/igab046.2732

**Published:** 2021-12-17

**Authors:** Donna Jensen, Theresa Abah, Carol Sewell, Terrence Ranjo

**Affiliations:** 1 California State University, Sacramento, Sacramento, California, United States; 2 California State University, Sacramento, Sacramento State University, California, United States; 3 Sacramento State University, Sacramento, California, United States

## Abstract

The COVID-19 pandemic has disproportionately impacted older adults, and the educational service-learning opportunities available to gerontology students. As an applied major, Sacramento State University’s Gerontology Department heavily depends on service-learning. The pandemic affected existing gerontology placements and their ability to host student learning. In addition, at the outset of the pandemic, Sacramento State University immediately terminated in-person service learning. The Gerontology Department prioritized student and community safety while still valuing the need for students to have meaningful and relevant community-based learning experiences. Gerontology faculty worked with community partners to shift these vital learning experiences. This poster presentation focuses on the creative ways the department engaged community partners to continue quality learning opportunities for students while assisting community partners with the unrelenting shifts in operations. Three innovative service-learning and community engagement practices will be addressed, including a) Partnering with the California Office of Emergency Services (CalOES) to create and provide the statewide Social Bridging Project for older adults throughout California; b) Expanding the relationship with Sacramento State’s Renaissance Society, a lifelong learning and community engagement program for older adults; and c) Partnering with a community-based Cardio-vascular Wellness Program to keep older adults active and engaged while remaining at home. The poster will review the pivot to virtual service learning and share how this shift enhanced student learning and community service. This will include expanding the use of technology and capitalizing on innovative methods to reach out and provide service to older adults, the local community, and the state of California.

